# Patient-reported oral mucositis in solid tumour patients undergoing chemotherapy: a Ugandan experience

**DOI:** 10.3332/ecancer.2023.1536

**Published:** 2023-04-21

**Authors:** Adriane Kamulegeya, Charles Mugisha Rwenyonyi, Jackson Orem

**Affiliations:** 1 School of Dentistry, Makerere University College of Health Sciences, Kampala, Uganda; 2 Uganda Cancer Institute, Kampala, Uganda

**Keywords:** mucositis, oral mucositis, chemotherapy adverse effects, OMDQ MTS

## Abstract

**Purpose:**

The mitotic rate of the gastrointestinal tract (GIT) mucosa predisposes the entire system to chemotherapeutic-induced mucositis but the oral cavity due to its accessibility provides an opening to evaluate the extent of the problem much more easily. In addition, the oral cavity being the gateway to the GIT affects the feeding ability of the patient when the ulcers set in.

It is therefore from this perspective that we embarked on a study to evaluate the extent of mucositis among patients being treated for solid tumours at our centre.

**Methods:**

Using the mouth and throat soreness (OMDQ MTS) questionnaire, we prospectively evaluated mucositis among 100 patients undergoing chemotherapy for solid tumours at the Uganda Cancer Institute. In addition to patient reported outcomes, we also had clinician assessed mucositis measurements.

**Results:**

Approximately, 50% of the participants were breast cancer patients. The results demonstrated that patient assessment of mucositis is possible in our setting at a 76% full compliance rate. Up to 30% of our patients reported moderate-to-severe mucositis, though the figure was lower as assessed by the clinicians.

**Conclusions:**

The self-reported OMDQ MTS can be useful in our setting for daily mucositis evaluation, hence leading to timely hospital visits before the manifestation of severe complications.

## Introduction

The mitotic rate of gastrointestinal mucosa along its entire spectrum makes it vulnerable to the effects of both chemotherapy and radiotherapy [1]. Mucositis lesions can present in the oral cavity, oropharynx and hypopharynx as ulceration and/or pseudomembranous formations. Some of the effects of oral mucositis (OM) include severe pain, interference with food intake and difficulty in speech. When severe, they can lead to dose limitation and interruptions in treatment [2].

OM is very common in patients treated for oral cavity and/or oropharynx tumours using radiotherapy due to the direct exposure of the oral mucosa, but the incidence is less among patients on chemotherapy alone [3]. Chemotherapy-induced OM, unlike radiotherapy-induced OM, is a continuum of gastrointestinal mucositis, but non-oral lesions are not so apparent thus the true incidence of mucositis is likely attenuated.

Unfortunately, even in cases where direct observation of mucositis is possible, measuring it is difficult and the actual implications on the patient cannot be judged based on just size and location. This makes both objective and subjective methods for recording the severity of mucositis difficult. It is not surprising that different centres use numerous tools and scales for recording mucositis. These tools and scales vary from patient-reported outcome measurement scales to clinician-based ones. Fortunately, reports have shown good agreement between mucositis severity as assessed by clinicians and patient self-reported measures. However, this is not always the case, especially if mucositis involves sites that are not visually accessible to the clinicians [4–6].

Additionally, differences in clinical scoring of mucositis using similar tools have been reported as a challenge and as such can lead to inconsistent estimates of the risk and severity [7]. Similar chemotherapeutic regimens for cancer among different/even the same patients, but using different tools, have reported divergent incidences and severity of mucositis [7–9].

Recently, coronavirus disease (COVID-19) came with its challenges in both evaluation and management of mucositis in both pre, during, and post-chemotherapy stages. These challenges may offer innovations such as using images taken by patients’ relatives in evaluating the incidence and severity of OM though with limitations [10].

Some studies, especially from Africa, have utilised retrospective analysis of mucositis risk and severity, but potential recall bias limits the usefulness of such studies [11]. It has been reported that even retrospective data collected in a clinical trial setting still has some challenges in grading and drawing definitive conclusions [12]. Therefore, prospective evaluation is more reliable.

Although some studies have looked at the impact of mucositis on patients’ therapeutic experiences, more work needs to be done especially in the context of developing countries where access to preventive and relieving agents is not readily accessible.

Overall, mucositis is a very important adverse effect of chemotherapy that must be addressed to reduce the burden of cancer, patients and their caregivers go through. It is worth noting that there is a huge financial strain on both the personal and public envelope that mucositis brings, and as such, it is a genuine public health issue that affects resources available for other health care and nutritional needs [13, 14]. Estimation of the true cost of the burden is hard to quantify due to the varying reporting and scoring methods, the perspective from which the problem is looked at, and the challenges of close daily evaluation of out-patients to mention, but a few [12, 14], hence outside the scope of the present study. To circumvent these challenges, the present study was aimed at a prospective follow-up of self-reported outcomes of chemotherapy-induced mucositis among patients with solid tumours attending the Uganda Cancer Institute (UCI). In addition, we evaluated the potential applicability of a self-reported tool in mucositis assessment in our population.

## Materials and methods

The study was conducted at the solid tumour centre ward and the outpatient department of UCI as part of a bigger study focusing on mucositis. The UCI is a 600-bed referral hospital in Uganda and a Center of Excellence for East Africa [15].

In this phase of the study after explaining the study and obtaining consent, we accessed the medical records of the recruited patients using a study-specific tool to get baseline data and information on some potential risk factors. At this stage, we also examined the patient and noted any oral mucosal lesions before any chemotherapeutic agents were administered.

This was an observational follow-up study aimed at developing a clinical factors model and also establishing the incidence of OM within the first 21 days among solid tumour patients who had received their first dose of chemotherapy. In addition, we wanted to find out the potential for using the patient-reported outcomes as a way of assessing mucositis.

The English or Luganda version of the OMDQ *mouth and throat soreness* (MTS) questionnaire was given to patients to record their daily experience and functional changes, with assistance from their trusted associates for those who could not read or write. This tool was filled from day 1 post-chemotherapy to day 14. Patients were asked to come back on the 3rd,7th, 10th and 14th day for assessment visits to evaluate levels of mucositis by nurses and clinicians who underwent multimedia-assisted face-to-face training. The examination was based on a uniform step-by-step algorithm for OM assessment (including evaluation of eight specified oral sites for erythema or ulceration) and case evaluation exercises. As per the WHO assessment, grade 0 = no mucositis; grade 1 = pain and erythema; grade 2 = ulceration, able to eat solid food; grade 3 = ulceration, able to consume only liquids; grade 4 = ulceration, inability to eat requiring tube or parenteral feeding. The 4-day scheduled visits were adopted from Stiff *et al* [26] with modifications to test the reliability and validity of the patient self-administered questionnaire in assessing the impact of OM on daily experience and functioning. These visits enabled the investigators to see and encourage the patient to fill out the questionnaire on daily basis. On the day 7 visit, in addition to the examination, a study clinician filled out another OMDQ MTS-C questionnaire with the patient to evaluate the test and retest reliability. The Luganda version of the OMDQ MTS had been earlier tested [16].

The reason for using self-reported assessment is that daily or frequent clinical evaluation of OM is impractical among all patients as they are often discharged after chemotherapy. While all clinical assessment scales require a clinical visit, self-reported OMDQ MTS can allow patients to observe the clinical progression of OM daily. However, to ensure quality, we had four assessment visits so that trained clinicians evaluated mucositis using the WHO oral assessment guide. To achieve a high, consistent quality OM assessment, nurses and clinicians underwent multimedia-assisted face-to-face training.

### Sample size calculations and statistical analysis

Given an incidence of 30% for grades 3 and 4 mucositis among South African patients undergoing chemotherapy [11], we used that figure, at a power of 80% to detect the incidence of patients reporting moderate-to-severe mucositis and factored in a 10% loss to follow up. Based on the above, we recruited 100 patients for the incidence arm.

The patients were broadly categorised into those who developed moderate-to-severe mucositis and those without too minor as per WHO assessment guidelines. Moderate-to-severe mucositis was defined as any score that was 2 and above as per the WHO assessment. This was a slight modification as described by Raber-Durlacher *et al* [17].

The area under the curve (AUC) plotting and testing was applied between the OMDQ MTS scale values and the clinician-determined WHO mucositis scores. Cohen’s kappa values and Spearman’s rank correlation coefficients were applied to appraise the strengths of association between the patients and clinician-administered OMDQ MTS on day 7 of the mucositis verification visit.

The predictive model was obtained by feeding in factors from analysis of variance (ANOVA) into binary regression. Other factors known to influence OM though non-significant in ANOVA analysis were also included in the binary analysis. Statistical analysis was done using Statistical Package for the Social Sciences 15 (IBM SPSS, Chicago, IL, USA).

### Ethical considerations

The study protocol was approved by the Higher Degrees and Ethics Committee of Makerere University School of Health Sciences, UCI Research and Ethics Review Board, and Uganda National Council of Science and Technology. The study participants gave written informed consent before recruitment into the study.

## Results

Out of 100 patients enrolled between February 2018 and July 2019, 25% were male and the demographic distribution of the study population is as shown in Table 1. Out of the recruited participants, 94% completed the required clinical evaluation visits and thus were considered evaluable for OM. The distribution of the study sample as per chemotherapeutic and cancer type are shown in Table 2. According to the WHO clinician assessment, 19 (20.21%) of the patients developed moderate-to-severe mucositis while 14 (14.89%) had grade 1 mucositis within the first 14 days of the 21-day follow-up period after their first dose of chemotherapy. On the other hand, based on the OMDQ MTS scale, 29 (30.9%) had moderate-to-severe mucositis.

### Feasibility

**Figure 1.**
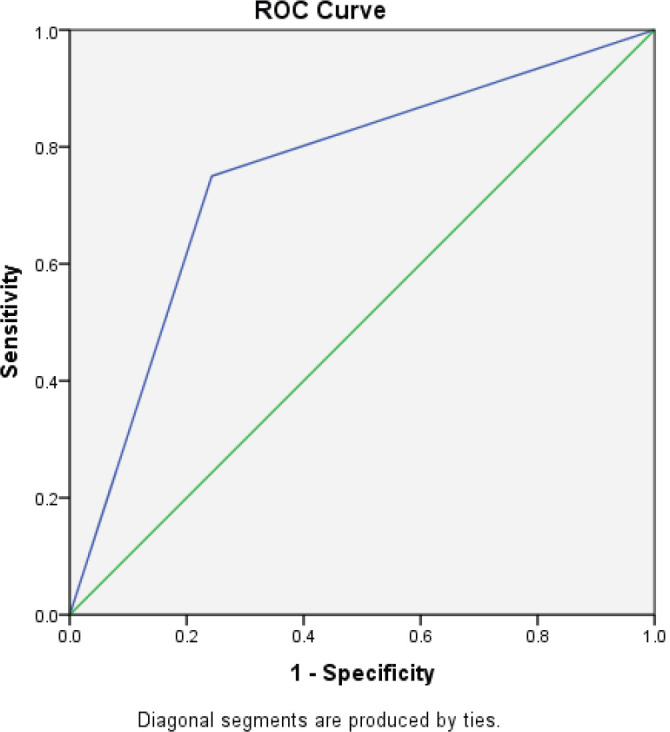
The AUC curve of self-reported mucositis against WHO mucositis score assessment.

Of the 94 patients who had all the scheduled clinical OM assessments, 88.30% filled at least half of the daily assessments of OMDQ MTS over the entire study period. Up to 76.6% filled all the daily OMDQ MTS. As per the OMDQ MTS, 29 (30.9%) of the patients had moderate-to-severe OM. The Cohen’s kappa values between patient-reported mucositis and clinician-assessed ones were minimal at 0.39 *p* = 0.001. The AUC was 0.75 (CI 0.62–089) *p* = 0.002 (Figure 1). The level of reproducibility on the important aspects of the OMDQ MTS was variable (Table 3).

### Association of OM with different variables

There was no significant difference between patients above 45 years and those below, those with and without a history of tobacco use in terms of incidence of moderate-to-severe OM (*p* = 0.37 and *p* = 0.67, respectively). Chemotherapy regimens that included 5-FU were significantly associated with the occurrence of severe-to-moderate OM (*p* = 0.04). Neither pre-chemotherapeutic haemoglobin nor neutrophil levels were statistically associated with the occurrence of OM (*p* = 0.53 and *p* = 0.74, respectively). The weight and surface area of the patients were also not significantly associated with increased occurrence of moderate-to-severe OM (*p* = 0.39 *p* = 0.15, respectively). Likewise, the oral hygiene status of the patients did not significantly affect the incidence of moderate-to-severe OM in the study population (*p* = 0.09).

From the binary logistic regression performed to ascertain the effects of 5-FU as part of the regimen, antimetabolites in the regimen, chemotherapy regimen, smoking history, age, weight, gender, surface area and cancer type on the likelihood that participants got moderate-to-severe mucositis. The logistic regression model was statistically not significant, *χ*2 (16) = 20.526, *p* = 0.197. Yet the model explained 43.6% (Nagelkerke *R2*) of the variance in mucositis and correctly classified 81.8% of the cases. Attempts to remove factors did not change the outcome.

### Time of OM appearance

**Figure 2.**
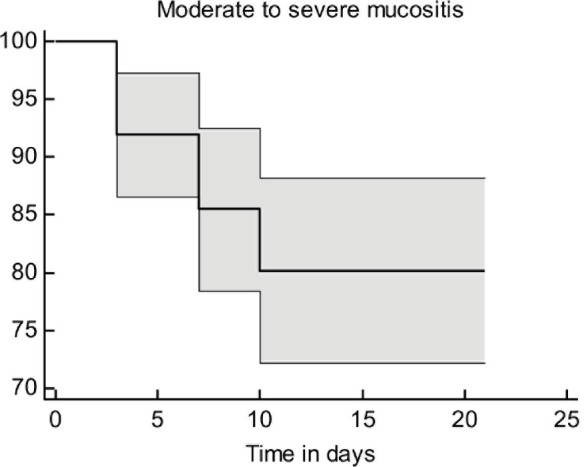
The survival curve for moderate-to-severe OM.

**Figure 3.**
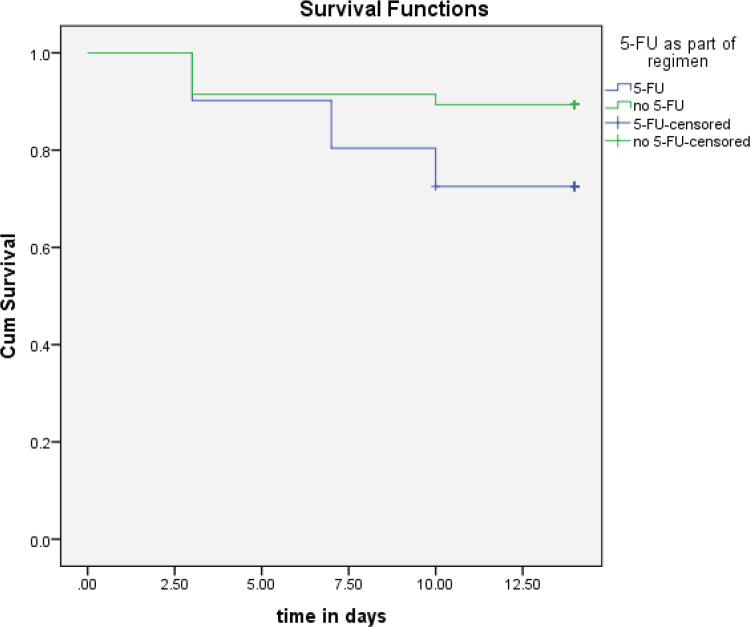
The time to occurrence of OM between groups that had 5-FU as part of the regimen compared to those who did not.

In the present study, patients had moderate-to-severe OMs within the first 3–10 days of receiving treatment. The survival probability for moderate-to-severe OM decreased with time post-chemotherapy (Figure 2). It is worth noting that the inclusion of 5-FU in the chemotherapy regimen was significantly associated with the time of appearance of moderate-to-severe OM signs (*p* = 0.05, Figure 3).

## Discussion

OM, as a complication of cancer chemotherapy, is a common phenomenon and can be highly morbid by severely interfering with alimentation. Often in Sub-Saharan Africa, we concentrate on the medication to treat the disease and clinical outcomes without paying attention to the side effects and their influence on compliance and non-physiological outcomes [18]. However, with globalisation and democratisation of information enabled by the internet and mobile phone penetration, patients are demanding better services, and as such, we must give due attention to the debilitating side effects of treatment [19]. It was on this basis that the present study was conceived. The study’s main aim was to establish the extent of the problem but also to evaluate the feasibility of monitoring OM in a Ugandan setting.

The present study employed both English and Luganda versions of the OMDQ MTS for self-assessment of OM and its impact on daily experience. Just like other authors have shown, the OMDQ MTS [20, 21] can be translated into many other local languages and become very useful in the assessment and facilitation of timely observations of OM and its progression. An A-76.6% full compliance rate was attained, indeed the present study has demonstrated that OMDQ MTS is a feasible tool in Sub-Saharan African settings for self-monitoring of changes in OM severity by adult patients. Furthermore, both self-report OMDQ MTS-C and the WHO clinician-based assessments were used, which increased the generalisability of the findings.

The findings in the present study are consistent with a previous study [8] from South Africa in which the incidence of self-reported OM among solid tumour patients receiving chemotherapy was moderately high. It was also similar to the incidence seen in the control group of an Egyptian randomised control trial [22]. Despite the patient-reported mucositis being higher than the clinician-evaluated one that used the WHO scale, it is worth noting that other studies have reported higher incidences and severity using patient-reported outcomes compared to clinician-assessments. An argument can be made that a single ulcer can be very disquieting to the patient, yet the clinician will capture it as one ulcer seen at a given anatomical site hence giving a lower grade. Additionally, the patient may experience symptoms much earlier than the time visible mucositis is detected by the clinicians [23].

One of the key challenges of measuring mucositis is the need for clinic visits, which are not only impractical for patients in poor settings but also an added burden to an already overstretched health workforce. Therefore, the idea of home-based self-assessment of adverse effects is of great value in capturing the consequences of cancer chemotherapy. Fortunately, in the present study, 76.6% filled all 15 days of daily OMDQ MTS comparing favourably with the 78% reported by Stiff *et al* [23]. In the present study, although the level of reproducibility on the important aspects of the OMDQ MTS was variable (Figure 1 and Table 3), they are comparable with findings from other similar studies [9, 23, 24].

In the present study, the inclusion of 5-FU in the chemotherapy regimen was the only statistically significant factor associated with an increased incidence of moderate-to-severe OM, this was not seen with other antimetabolites or factors. The effects of 5-FU on our patient population are as reported in other published reports [25].

## Conclusions and recommendations

The present study observed that OM is common among patients receiving their first cycle of chemotherapy for solid tumours in the Ugandan population. Although the response rate in this study was good, more work is required to determine the psychometric properties of the OMDQ MTS, especially in diverse Sub-Saharan African populations. There is also a need to find out the relevance of agreement or lack of it between OMDQ MTS and clinician-based OM assessment scales. The huge variations in chemotherapy regimens, burden, and cost of frequent clinical assessments of OM, necessitate a subcontinental multi-institutional collaborative research with larger population groups to tease out the effect of other reported factors that influence the toxicity risk of solid tumour chemotherapy.

## Conflicts of interest

None declared.

## Funding

This research was supported by a NURTURE grant a D43 grant, grant number D43TW010132. The funder provided funds for patient compensation.

## Acknowledgment of research support

Funding support for this study was provided by NURTURE a D43 grant, grant number D43TW010132. The funding covered the salary of the research assistants and the travel and compensation expenses required for data collection.

## Figures and Tables

**Table 1.** The frequency distribution of the participants according to demographic characteristics (*n* = 100).

Variable	All	Gender	
		Male (*n* = 25)	Female (*n* = 75)
Age range (Mean ± SD)	24–79 (45.3 ± 11.9)	29–79 (49.4 ± 12.9)	24–75 (44.0 ± 11.5)
Surface area *M*^2^ range	1.2–2.2 (1.62 ± 0.16)	1.22–1.80 (1.60 ± 0.13)	1.20–2.20 (1.64 ± 0.17)
Weight (kg)	35–111 (60.02 ± 13.01)	42–75 (56.88 ± 8.84)	36–111 (60.66 ± 14.39)
Haemoglobin level (g/dl)	5.53–16.40 (12.18 ± 2.14)	5.53–15.50 (11.84 ± 2.07)	7–16.40 (12.19 ± 2.24)

**Table 2.** The frequency distribution of participants according to the type of cancer and chemotherapeutic regimen (*n* = 94).

Type of cancer	Chemotherapy regimen	*N* (%)
Cancer of breast (*n* = 49)	Cyclophosphamide, Adriamycin, 5-FU	24 (25.55%)
	Cyclophosphamide, Adriamycin	1 (1.06%)
	Cyclophosphamide, 5-FU	1 (1.06%)
	Cyclophosphamide, Dexamethasone,5-FU	1 (1.06%)
	Cyclophosphamide	1 (1.06%)
	Cyclophosphamide, Doxorubicin, 5-FU	13 (13.83%)
	Cyclophosphamide, Doxorubicin	2 (2.13%)
	Cyclophosphamide, Doxorubicin, 5-FU, Leucovorin	1 (1.06%)
	Cyclophosphamide, Methotrexate	3 (3.19%)
	Cisplatin, Leucovorin, 5-FU	1 (1.06%)
	Cisplatin, Leucovorin	1 (1.06%)
Cancer of oesophagus (*n* = 8)	Paclitaxel	7 (7.45%)
	Doxorubicin	1 (1.06%)
Cancer of stomach (*n* = 7)	Oxaliplatin, Capecitabine	3 (3.19%)
	Cisplatin, Docetaxel	3 (3.19%)
	Cisplatin, 5-FU	1 (1.06%)
Cancer of rectum (*n* = 6)	Oxaliplatin, Capecitabine	4 (4.26%)
	Oxaliplatin, Leucovorin, 5-FU	1 (1.06%)
	Irinotecan, Leucovorin, 5-FU	1 (1.06%)
Hepatocellular carcinoma (*n* = 5)	Doxorubicin	2 (2.13%)
	Adriamycin	3 (3.19%)
Epidemic Kaposis sarcoma (*n* = 3)	Paclitaxel	3 (3.19%)
Cancer of colon (*n* = 5)	Oxaliplatin, Capecitabine	5 (5.32%)
Cancer of pancreas (*n* = 3)	Gemcitabine, Oxaliplatin	3 (3.19%)
Cancer of cervix (*n* = 2)	Cisplatin, Paclitaxel	1 (1.06%)
	Cisplatin	1 (1.06%)
Cancer of thyroid	Cisplatin, Doxorubicin	1 (1.06%)
Monophasic synovial sarcoma	Doxorubicin, Dacarbazine	1 (1.06%)
Non specified Soft tissue Sarcoma	Doxorubicin, Dacarbazine	1 (1.06%)
Carcinoma NPC	Cisplatin, 5-FU	1 (1.06%)
Cancer of penis	Cisplatin, Paclitaxel, Magnesium Sulphate	1 (1.06%)
Cancer of lung	Cisplatin Etoposide	1 (1.06%)

**Table 3.** The level of agreement between patient self-reported mucositis to clinician-administered OMDQ MTS-C (*n* = 88).

	Self-administered versus clinician on day 7					
	Level of mouth and throat soreness in past 24 hours	Limitation on swallowing past 24 hours	Limitation on drinking past 24 hours	Limitation on eating past 24 hours	Limitation on talking past 24 hours	Limitation on sleeping past 24 hours
Cohen’s kappa value	0.46	0.44	0.42	0.55	0.47	0.27
Spearman’s rank correlation coefficient	0.69	0.54	0.61	0.66	0.68	0.46
